# Effects of D-Chiro-Inositol on Glucose Metabolism in db/db Mice and the Associated Underlying Mechanisms

**DOI:** 10.3389/fphar.2020.00354

**Published:** 2020-03-26

**Authors:** Chunxue Fan, Weishi Liang, Min Wei, Xiangbo Gou, Shuying Han, Jing Bai

**Affiliations:** ^1^School of Pharmacy, North China University of Science and Technology, Tangshan, China; ^2^Clinical Medical College, North China University of Science and Technology, Tangshan, China; ^3^School of Basic Medical Sciences, North China University of Science and Technology, Tangshan, China

**Keywords:** D-chiro-inositol, type 2 diabetes mellitus, db/db mice, glucose metabolism, insulin resistance

## Abstract

In this study, we observed the effect of D-chiro-inositol (DCI) on glucose consumption in type 2 diabetic db/db mice, and investigated the relevant mechanism. We discovered that the stability of 24-h blood glucose under the nonfasting condition and decreased glucose tolerance were both alleviated after treatment with DCI. Moreover, the content of glycosylated protein and advanced glycation end products in the serum was reduced, the damage in the liver tissue was alleviated, and the synthesis of liver glycogen was significantly promoted. In addition, DCI increased the expression of insulin receptor substrate 2 (IRS2), phosphatidylinositol 3-kinase (PI3K), protein kinase B (AKT), glucose transporters 4 (GLUT4), and phospho-AKT (S473) protein. In contrast, DCI decreased the expression level of glycogen synthase kinase 3β (GSK3β) protein in liver tissue to various degrees, as shown by immunohistochemistry and western blotting. Furthermore, DCI increased the mRNA expression of IRS2, PI3K, AKT, and GLUT4, and reduced that of GSK3β in liver tissue, as demonstrated by polymerase chain reaction. Finally, DCI promoted glucose consumption in high glucose-stimulating HepG2 cells and increased the expression of IRS2 protein in HepG2 cells, as revealed by fluorescence staining and flow cytometry. Our results indicate that DCI can significantly improve glucose metabolism in diabetic mice and HepG2 cells. This effect may be associated with the upregulation of IRS2, PI3K, AKT, and GLUT4 and downregulation of GSK3β.

## Introduction

Diabetes mellitus is a lifelong metabolic disease characterized by hyperglycemia, which is caused by insufficient secretion or dysfunction of insulin (Hummel et al., 1966; Rutter et al., 1999; Tian et al., 2017). At present, the incidence of diabetes is increasing annually. The prevalence of diabetes in China is approximately 11%, ranking first worldwide (Cheung et al., 2018). As one of the independent risk factors of type 2 diabetes mellitus (T2DM), insulin resistance (IR) is closely associated with various metabolic diseases (Cordain et al., 2003; Mehanna et al., 2018). In addition, IR induces various diabetic complications (e.g., diabetic liver injury) (Ji et al., 2009), which occur throughout the entire developmental process of T2DM and cause harm to diabetic patients. Therefore, it is of great importance to identify effective drugs for the prevention and reduction of IR.

D-chiro-inositol (DCI) is one of the nine isomers of inositol, which has optical rotation and is present in plants (e.g., buckwheat and soybean) (Steadman et al., 2000; Song et al., 2016). This type of inositol is frequently used as a dietary health supplement in the clinical treatment of T2DM (Gao et al., 2016b), promoting liver lipid metabolism and possessing other physiological functions (Ken-Ichi et al., 2006; Whiting et al., 2013). It has been reported that, as an insulin sensitizer, the structure of DCI is similar to that of pH2.0 insulin regulatory mediators. The latter is an important component of insulin's second messenger phosphoinositol glucan, which may promote lipid metabolism in liver and insulin signal transduction (Larner et al., 2010). Furthermore, DCI may significantly increase glucose consumption in IR-HepG2 cells and significantly reduce their IR (Gao et al., 2016a).

However, now the molecular mechanism isn't very clear, still needs to be improved. Therefore, in this study, we further investigated the molecular mechanism through the entire IRS2/PI3K/AKT/GSK3β/GLUT4 signaling pathway. In the present study, an intervention with DCI in db/db mice and high glucose-stimulating HepG2 cells was used to observe its effect on glucose metabolism *in vivo* and *in vitro*. Moreover, the possible mechanism from the insulin signal transduction pathway was investigated, which may provide an experimental basis for further research regarding the clinical applications of DCI.

## Methods

### Animals and Treatments

Male db/db mice (10-week-old, 36–45 g) and male db/m mice (10-week-old, 17–20 g) in C57BLKS/J (BKS) inbred lines were purchased from Changzhou Cavens Laboratory Animal Co. Ltd. (Changzhou, China) (License key: SCXK (Su) 2016-0010). ^60^Co radiation mice granule feedstuff was purchased from Nanjing Beisifu Feed Co. Ltd. (Nanjing, China) (Lot number: 20171050001MF01). The animal experiments were performed in specific-pathogen-free barrier laboratory at the Experimental Animal Center of North China University of Science and Technology (Tangshan, China).

Following adaptive feeding for 1 week, blood was drawn from the tails of the mice to determine the level of blood glucose using a blood glucose meter (Abbott, USA). Ten male db/m mice were selected to form the normal control group (NCG), while 30 db/db mice were selected based on the levels of random blood glucose (RBG) and blood glucose after 2 h of oral glucose load. Subsequently, the 30 db/db mice were randomly divided into three groups receiving different treatments: high-dose DCI group (HDCIG), low-dose DCI group (LDCIG) and model control group (MCG). The HDCIG and LDCIG mice received DCI (purity: > 98.0%, Lot number: I0632, TCI, Japan) at a dose of 70 mg·kg^-1^·d^-1^ and 35 mg·kg^-1^·d^-1^ by gavage (gavage volume is 0.1 ml/10g), respectively. Meanwhile, the NCG and MCG mice received an equal volume of pure water. All treatments were administered at 9 a.m. every day, and the duration of the entire experiment was 6 weeks.

### Measurement of Weight and Concentration of Blood Glucose

Daily water intake and feed intake were measured every three days, and the weight of each group was measured at 9:00 in every week. After 3 weeks of treatment, the concentration of glucose was measured after 10 h of fasting using the oral glucose tolerance test (OGTT). The dose of glucose used for the OGTT was 2g·kg^-1^. The area under the curve (AUC) was calculated according to the level of blood glucose and the trapezoidal area summation method, wherein G0, G30, G60, and G180 are blood glucose levels at different time points (Bao et al., 2016). AUC=15(G0+G30) + 15(G30+G60) + 30(G60+G120). After 6 weeks of administration, changes in the 24-h blood glucose under nonfasting conditions were measured at 4:00, 8:00, 12:00, 16:00, 20:00, and 24:00 (0:00).

### Measurement of Serum Biochemical Indices

Twenty-four hours after the last administration, blood samples were collected from the eye socket after anesthetized with isoflurane inhalation, and the serum was separated. Insulin (INS) and levels of advanced glycation end products (AGEs) in the serum were measured using the enzyme-linked immunosorbent assay (ELISA) kit (Cat number: JYM0351Mo and JYM0171Mo, Wuhan Colorful Gene Biological Technology Co. Ltd., China) according to the instructions provided by the manufacturer. The level of glycosylated serum protein (GSP) was measured using the GSP assay kit (Cat number: A037-2, Nanjing Jiancheng Bioengineering Institute, China).

### Liver Sampling and Microscopical Examination

The liver tissue was removed immediately after the mice were sacrificed, and rinsed with cool saline water. Weigh the liver and calculate the liver index (LI, LI = liver weight/body weight×100%).

In addition, the left lobe of the liver was divided into two portions and stored for Western blotting and polymerase chain reaction (PCR) analyses. Following the partial cutting of the intact liver tissue, it was soaked in 4% polyoxymethylene solution, and subsequently subjected to conventional paraffin embedding. Finally, the liver tissue was cut into 3.5-μm-thick sections. After periodic acid-Schiff (PAS) staining using a glycogen staining kit (Cat number: D004, Nanjing Jiancheng Bioengineering Institute, China), the histological structure of liver tissue was observed under a BX50 microscope (Olympus, Japan). The level of hepatic glycogen was measured using Liver/Muscle glycogen assay kit (Cat number: A043-1-1, Nanjing Jiancheng Bioengineering Institute, China).

### Immunohistochemistry

Immunohistochemistry was performed using a universal two-step test kit (Cat number: PV-9000, ZSGB-BIO, China). Paraffin-embedded sections of liver tissues were dewaxed, hydrated, and boiled to repair antigen through ethylenediaminetetraacetic acid high-pressure heating. Following a wash with phosphate-buffered saline (PBS), the sections were soaked with serum (Cat number: ZLI-9022, ZSGB-BIO, China) and incubated with the following primary antibodies: anti-IRS2 (1:200, Cat number: 3089S, Cell Signaling Technology, USA), anti-phosphatidylinositol 3-kinase (anti-PI3K) (1:200, Cat number: YT3711, ImmunoWay, USA), anti-protein kinase B (anti-AKT) (1:200, Cat number: ab179463, Abcam, UK), anti-phospho-AKT (anti-P-AKT) (S473) (1:200, Cat number: ab81283, Abcam, UK), anti-GLUT4 (1:300, Cat number: BM2162, Boster, USA) and anti-glycogen synthase kinase 3β (anti-GSK3β) (1:250, Cat number: YT2082, ImmunoWay, USA). Following storage in a refrigerator at 4°C overnight, the sections were incubated with the secondary antibody in the kit at 37°C. After washing with PBS, the sections were dyed using 3,3'-diaminobenzidine (Cat number: ZLI-9018, ZSGB-BIO, China) for 50 s. The nucleus was stained with hematoxylin for 1 min. In the negative control group, PBS was used instead of primary antibody to perform the aforementioned experiments. Finally, the positive-signal proteins were colored brownish yellow (Gartmann et al., 2018), and the expression and distribution of related proteins were observed under a light microscope (OLYMPUS BX5, Japan). The average integral absorbance was determined using the Image-Pro Plus 6.0 software.

### Western Blotting Analysis

The liver tissue was cut into small pieces on ice and subsequently homogenized in protein lysate buffer (Cat number: PS0013, Leagene, China). The supernatant was obtained after centrifugation at low temperature, and the protein was quantified using a bicinchoninic acid assay kit (Cat number: PT0001, Leagene, China). Proteins were separated on gradient sodium dodecyl sulfate-polyacrylamide gel electrophoresis (Cat number: PE0017, Leagene, China) and electrophoretically transferred to polyvinylidene difluoride blotting membranes (Cat number: 10600023, GE, USA). The membranes were blocked with 5% nonfat dry milk for 2 h, and subsequently incubated with primary antibodies overnight at 4°C (Gou et al., 2018). The primary antibodies used were as follows: anti-PI3K (1:1,000), anti-AKT (1:1,000), anti-P-AKT (S473) (1:1,000), anti-GLUT4 (1:1,000), anti-GSK3β (1:500), and anti-β-actin (1:1,000, Cat number: AP0060, Bioworld, USA). After washing with TBST (Cat number: T1080, Solarbio, China), the membranes were incubated with goat anti-rabbit/anti-mouse fluorescently labeled secondary antibodies (1:5,000, 074-1506, and 074-1806, Seajet, China). Quantification of the signals was performed using the Odyssey Infrared Imaging System (Cat number: LICOR 9120, Li-COR, USA). The protein bands were normalized to the β-actin band in each sample.

### PCR Analysis

The mRNA expression levels of IRS2, AKT, PI3K, GLUT4, and GSK3β were determined through PCR. Total RNA was extracted from the liver of mice using Trizol (Cat number: 15596026, Thermo Fisher Scientific, USA), and the quality of the extracted RNA was confirmed using a NanoPhotometer-N50 ultra-microspectrophotometer (Implen, Germany) (Liang et al., 2018). Total RNA (5 μg) was retro-transcribed into cDNA using the RevertAid first strand cDNA synthesis kit (Lot number: 00422714, Thermo Fisher Scientific, USA). PCR was performed using 1.5 μl cDNA, 12.5 μl 2×Es Taq MasterMix (Dye), and 1.0 μl primers, in a total volume of 25 μl. PCR amplification was performed under the following conditions: initial activation of the hot-start DNA polymerase for 5 min at 95°C, followed by 34 cycles of step 2 (i.e., 95°C for 30 s, 56°C for 30 s, and 72°C for 40 s), and subsequently 72°C for 8 min before a constant temperature of 4°C. The amplified cDNA was separated through agarose gel electrophoresis, and quantification of the signals was performed by the automatic gel imaging analysis system (Cat number: 1708270, BIO-RAD, USA). The PCR primer sequences used are shown in **Table 1**.

**Table 1:** Primers of polymerase chain reaction (PCR) analysis for genes.

Primer	Primer sequence	Product length (bp)
M-ACTB-F	GTTGGTTGGAGCAAACATCCC	174
M-ACTB-R	TTAGGAGTGGGGGTGGCTTT	
M-IRS2-F	CCCGAGTCAATAGCGGAGAC	93
M-IRS2-R	ACAGTGGCTCAGGGGTCTAT	
M-PI3K-F	ATTGACAGTAGGAGGAGGTTGG	148
M-PI3K-R	CTTTCTGCGTCAGCCACAT	
M-AKT-F	TTCTATGGTGCGGAGATTGTGT	132
M-AKT-R	CAGCCCGAAGTCCGTTATCT	
M-GLUT-4-F	TTCCTTCTATTTGCCGTCCTC	170
M-GLUT-4-R	TCTGGCCCTAAGTATTCAAGTTCT	
M-GSK3-F	CTTTGGAGCCACTGATTACACG	134
M-GSK3-R	GGACCTTTATTATTTCCACCAACTG	

### Investigation of the Effect of DCI on the Viability of HepG2 Cells

DCI was diluted using Dulbecco's Modified Eagle Medium (Cat number: 31600, Solarbio, China) to a concentration of 4 mg/ml and stored at −80°C. HepG2 cells were supplied by the Shanghai Fudan University Cell Library (Shanghai, China). Subsequently, HepG2 cells were placed into 96-well plates (0.15 ml per well) and cultured for 24 h in a CB150 5% carbon dioxide culture box (BINDER, Germany) at 37°C. After discarding the culture medium, complete medium containing DCI in different concentrations (i.e., 0, 25, 50, 100, 200, 400, and 800 μg/ml) was added (each concentration was assayed in six wells). After cultivation for 24 h, 10-μl Cell Counting Kit-8 (CCK-8) was added into each well, and the culture plate was incubated in the incubator for 1 h at 37°C. The optical density (OD) value at 450 nm was determined using a multimode reader, and the OD value was proportional to the number of living cells. Cell viability in the control group was 100%, and cell viability in each group was calculated using the following formula:

Cell viability  (%) = (OD value of drug group/OD value of control group) × 100 %.

### CCK-8 for the Detection of Cell Viability in High Glucose-Stimulating HepG2 Cells

Under the aforementioned cell culture conditions, high-glucose medium (33.3 mmol/L and 55.5 mmol/L) was added for 48 h, and the supernatant was discarded. The high-glucose medium containing the corresponding DCI was added to each well for 48 h. After addition of CCK-8 (Dojindo Laboratories, Japan) for 1 h, the OD value was determined. At the same time, eight wells of normal cells containing normal culture Medium (glucose concentration: 22.2 mmol/L) were prepared. According to the results of this investigation, the optimal concentrations of DCI (i.e., 8, 16, and 32 μg/ml) were selected as the experimental concentrations.

### Glucose Oxidase Method for the Detection of Glucose Consumption in HepG2 Cells

Under the aforementioned cell culture procedures, the high-glucose medium (55.5 mmol/L) was added to the 96-well plate containing HepG2 cells for 48 h. After the supernatant was discarded, high-glucose medium containing different concentrations of DCI was added to each well. There were five groups of cells in this experiment: normal cells in normal medium (glucose concentration: 22.2 mmol/L), model cells in high-glucose medium (glucose concentration: 55.5 mmol/L), and cells in high-glucose medium containing different concentrations of DCI (i.e., 32, 16, and 8 μg/ml). Each group of cells was assayed in eight wells and cultured for 48 h. Subsequently, the concentration of glucose was determined using the glucose oxidase method, which required to 5 μl of medium from each well. Lastly, CCK-8 was added to each hole for 1 h and the OD value was measured. The formula used for the calculation of glucose consumption was as follows:

Glucose consumption= (glucose concentration in the medium at the timeof administration−glucose concentration in the medium48 h after administration  ).

### Fluorescence Staining

HepG2 cells in the logarithmic growth phase were collected, and their concentration was adjusted to 10^4^/ml using normal medium. The cells were placed in six-well plates at 5 ml per well (5×10^4^ cells). After the cells were attached, the medium was aspirated, and 5 ml of high-glucose medium (55.5 mmol/L) were added to the wells for 72 h. Subsequently, they were replaced with high-glucose medium (5 ml per well) containing DCI (32 μg/ml, 16 μg/ml) for 96 h. There were four groups in this experiment: normal cells in normal medium, model cells in high-glucose medium, and cells in high-glucose medium containing different concentrations of DCI (i.e., 32 or 16 μg/ml). Each group was assayed in two wells.

The cells were soaked in 4% paraformaldehyde at 4°C for 10 min. Subsequently, the cells were incubated with primary antibody (anti-IRS2, 1:500) at 4°C overnight. In addition, a fluorescent secondary antibody (Cat number: ab150073, abcam, USA, 1:500) was added to the cells. Then, 4′,6-diamidino-2-phenylindole was added to the cells, and the nuclei were stained for 5 min (Shridas et al., 2018). Finally, the cells were observed under a microscope (OLYMPUS BX5, Japan). The negative control group received PBS instead of primary antibody.

### Flow Cytometry

Under the cell culture condition and treatment administration described in Fluorescence Staining of this article, the cells were trypsinized. The trypsin reaction was terminated using PBS, and the cells were transferred to 5 ml of Eppendorf tubes. The cells were dispersed into single cells, centrifuged at 1,500 rpm for 5 min, and the supernatant was removed. After adding 2% paraformaldehyde for 10 min, the cells were centrifuged at 1,500 rpm for another 5 min and washed once with PBS. The cells of each group were incubated with primary antibody (anti-IRS2, diluted with 1% bovine serum albumin, 1:150) at 37°C for 45 min, and washed once with PBS. Subsequently, the cells of each group were incubated with a fluorescent secondary antibody (Cat number: ab150073, abcam, USA, diluted with PBS, 1:200, 37°C, 20 min), and resuspended in PBS, to reach a concentration of 10^6^/ml cells in each tube. The expression rate of positive cells was determined through flow cytometry (Becton Dickinson, USA). Cells in the negative control group were incubated with PBS instead of primary antibod.

### Statistical Analysis

Statistical analysis was performed using the SPSS 19.0 software, and the experimental data were expressed as means ± standard deviation (SD) or means ± standard error of the mean (SEM) (Soll et al., 2018). Statistical significance was determined using one-way analysis of variance, followed by a least signiﬁcant difference test for multiple comparisons. P < 0.05 denoted statistical signiﬁcance.

## Results

### *In Vivo* Study

#### Results of Changes in Daily Water Intake, Daily Feed Intake, Weight, and LI

**Figure 1:**
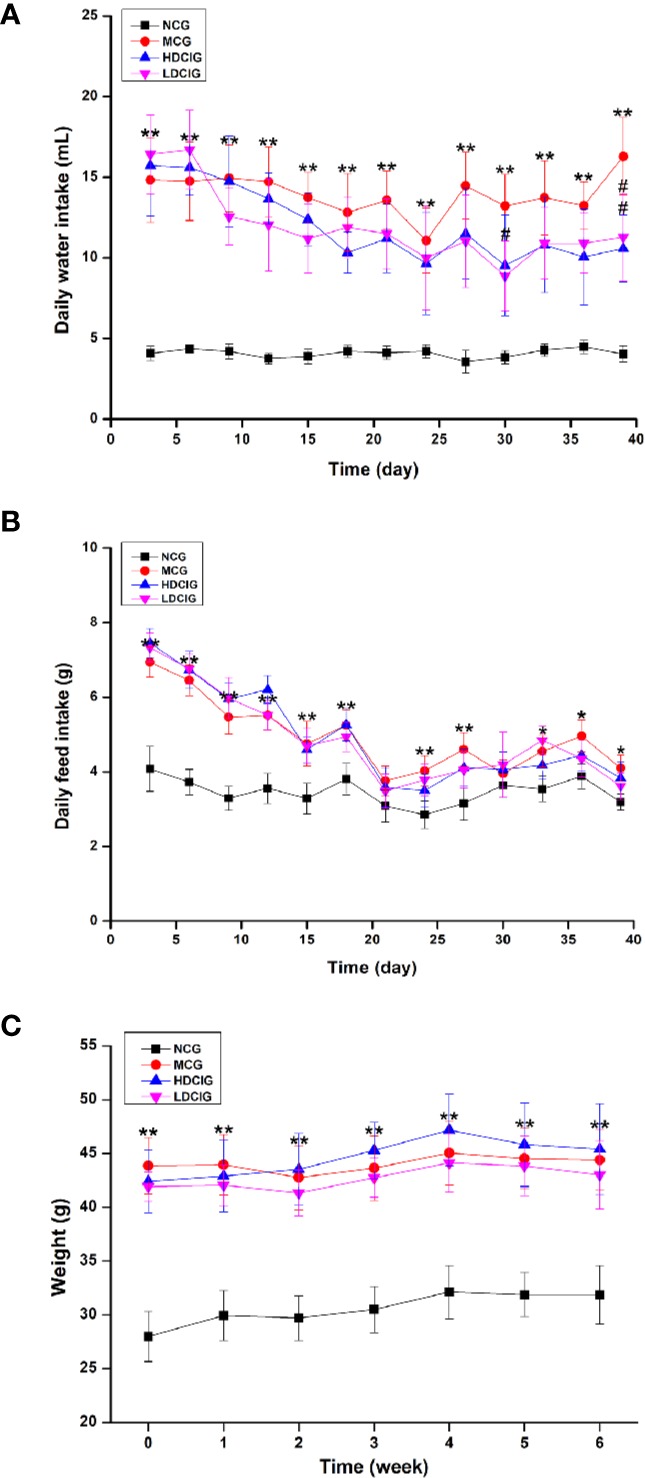
Results of changes in daily water intake, daily feed intake, and weight of db/db mice. **(A)** Daily water intake. **(B)** Daily feed intake. **(C)** Weight of db/db mice. All values are expressed as means ± SD, n=10. *P < 0.05, **P < 0.01 vs. NCG mice; ^#^P < 0.05 vs. model control group (MCG) mice.

In **Figure 1**, the levels of daily water intake and daily feed intake of db/db mice might be higher than those in db/m mice at each point in time (P < 0.01). Compared with MCG, daily water intake in administration groups gradually decreased with unvisible difference in daily feed intake. After 6 weeks of feeding, each group of mice gained weight, and there was no significant difference between MCG and administration groups. The level of LI in HDCIG was higher than that in MCG in **Table 2** (P < 0.05).

**Table 2:** Effects of D-chiro-inositol on body weight and liver index (LI) in db/db mice.

Group	Body weight (g)	Liver weight (g)	LI (%)
NCG	31.88 ± 2.720	1.785 ± 0.185	5.656 ± 0.914
MCG	44.4 ± 2.827**	2.462 ± 0.289**	5.539 ± 0.487
HDCIG	45.42 ± 4.225	2.137 ± 0.441^#^	4.736 ± 1.026^#^
LDCIG	43.022 ± 3.169	2.213 ± 0.271	5.140 ± 0.432

**P < 0.01 vs. normal control group (NCG); ^#^P < 0.05 vs. model control group (x ± SD, n=10).

#### Effects of DCI on the Level of Blood Glucose in db/db Mice

**Figure 2:**
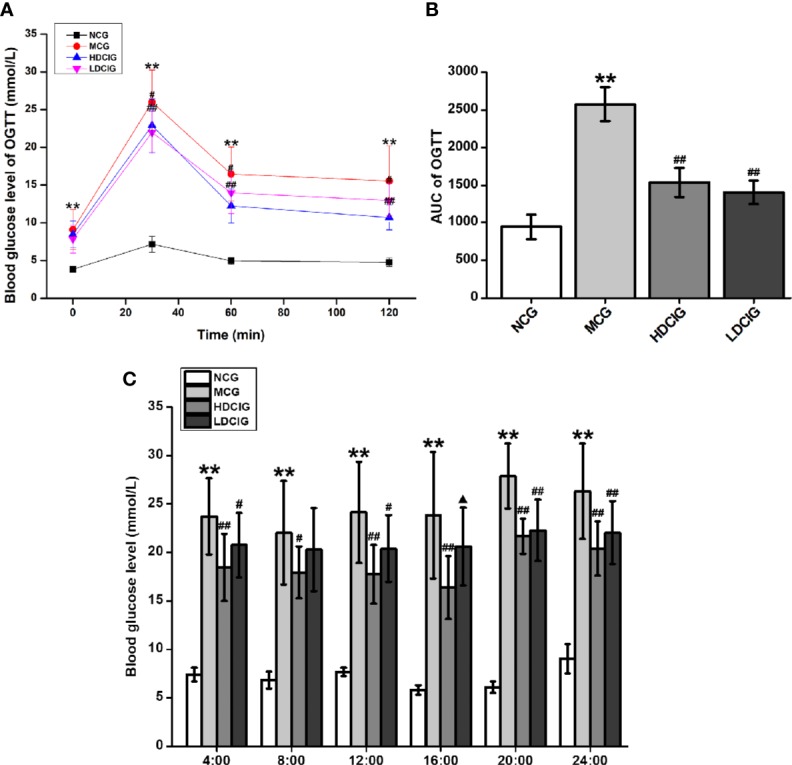
Effects of D-chiro-inositol (DCI) on blood glucose in db/db mice. **(A)** The level of blood glucose was determined in different groups after 3 weeks of administration using the oral glucose tolerance test (OGTT). **(B)** The area under the curve (AUC) of the OGTT is shown. **(C)** Dynamic changes in non-fasting 24 h blood glucose in each group of mice were determined after the 6-week administration period. All values are expressed as means ± SD, n=10. **P < 0.01 vs. normal control group (NCG) mice, ^#^P < 0.05, ^##^P < 0.01 vs. model control group (MCG) mice; ^▲^P < 0.05, vs. high-dose D-chiro-inositol group (HDCIG) mice.

The levels of blood glucose in the different groups after 3 weeks of DCI administration were determined using the OGTT (**Figure 2A**). The AUC derived from the OGTT is shown in **Figure 2B**. The AUC in the MCG was significantly higher than that observed in the NCG (P < 0.01). In addition, the AUC in the HDCIG and LDCIG was significantly decreased compared with that reported in the MCG (P < 0.01). Therefore, DCI decreased the levels of blood glucose in db/db mice.

Dynamic changes in the nonfasting 24-h blood glucose in each group were determined after 6 weeks of administration (**Figure 2C**). The levels of blood glucose in the NCG reached their highest value around 24:00 (P < 0.01). In the MCG, HDCIG, and LDCIG groups, the blood glucose was relatively higher around 20:00, and there was no significant difference compared with the blood glucose result at 24:00, which may be related to abnormal glucose metabolism in db/db mice. Notably, the levels of blood glucose in these groups were significantly higher than those reported in the NCG at each time point (P < 0.01). The levels in the HDCIG and LDCIG were all reduced to different degrees compared with those reported in the MCG.

#### Effects of DCI on the Levels of INS, GSP, and AGEs in the Serum of db/db Mice

**Figure 3:**
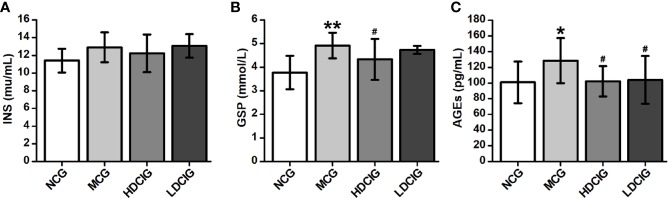
Effects of D-chiro-inositol (DCI) on serum indices in db/db mice. **(A)** The result of insuin (INS). **(B)** The result of glycated serum proteins (GSP). **(C)** The result of advanced glycation end products (AGEs). All values are expressed as means ± SD, n=10. *P < 0.05, **P < 0.01 vs. normal control goup (NCG) mice; ^#^P < 0.05 vs. model control group (MCG) mice.

There was no significant difference in the levels of INS between the groups (P > 0.05, **Figure 3A**). Of note, the levels of GSP in the NCG were lower than those observed in the other three groups (P < 0.05, **Figure 3B**). In addition, the highest level of GSP was noted in the MCG (P < 0.05). Moreover, the levels of AGEs in the MCG were higher than those reported in the other three groups (P < 0.05, **Figure 3C**).

#### Effects of DCI on the Synthesis of Hepatic Glycogen in db/db Mice

**Figure 4:**
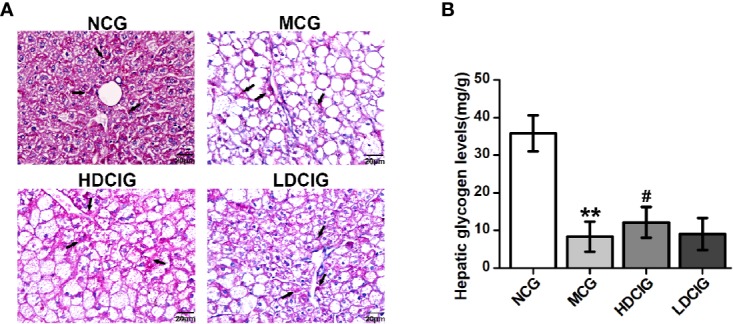
Results of PAS staining of liver tissues obtained from each group of mice. **(A)** Representative images of liver tissue stained with PAS (200×) are listed. Arrows indicate hepatic glycogen. **(B)** The hepatic glycogen levels of db/db mice are measured. All values are expressed as means ± SD, n=10. **P < 0.01 vs. (NCG) mice; ^#^P < 0.05 vs. model control group (MCG) mice.

The levels of hepatic glycogen were determined through PAS staining, in which the glycogen and other PAS-positive components in the liver tissue stained red (**Figure 4A**). The hepatocellular morphology of NCG was normal, the content of glycogen was higher, and the distribution of glycogen near the blood vessel was more. The structure of liver cells in the MCG was disordered that the cells were obviously swollen and deformed, and decreased levels of glycogen were observed (P < 0.01, **Figure 4B**). Compared with the MCG, the distribution of glycogen of HDCIG mice was relatively increased (P < 0.05).

#### Determination of the Effects of DCI on the Expression Levels of Insulin IRS2/PI3K/AKT/GSK3β/GLUT4 Signaling Pathway-Related Proteins in Liver Tissues Through Immunohistochemistry

**Figure 5:**
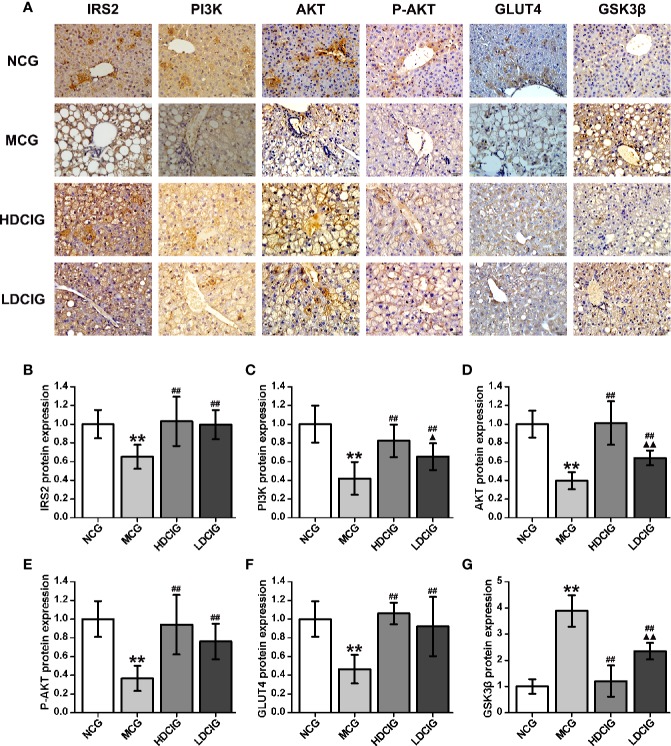
Results of immunohistochemistry for the determination of relative protein expression. **(A)** Representative immunohistochemical images of liver tissue regarding insulin receptor substrate 2 (IRS2), phosphatidylinositol 3-kinase (PI3K). protein kinase B (AKT), phospo-AKT (S473) (P-AKT), glucose transporters 4 (GLUT4) and glycogen synthase kinase 3β (GSK3β) (200×) are listed. **(B)** The result of IRS2 protein expression. **(C)** The result of PI3K protein expression. **(D)** The result of AKT protein expression. **(E)** The result of P-AKT protein expression. **(F)** The result of GLUT4 protein expression. **(G)** The result of GSK3β protein expression. The integrated optical density (IOD) of the immunohistochemistry-positive sites in the sample sections and the total area (Area) were determined using Image-Pro Plus 6.0. The mean optical density was calculated (MOD=IOD/Area). The results obtained for the different groups were normalized to those reported in the normal control group (NCG). All values are expressed as means ± SD, n=10. **P < 0.01 vs. (NCG) mice; ^##^P < 0.01 vs. model control group (MCG) mice; ^▲^P < 0.05, ^▲▲^P < 0.01 vs. high-dose D-chiro-inositol group (HDCIG) mice.

The results obtained for the different groups were normalized to those reported in the NCG (**Figure 5**). It was shown that representative pictures of paraffin-embedded liver tissue sections processed by immunohistochemical staining regarding IRS2, PI3K, AKT, p-AKT (S473), GLUT4, and GSK3-β proteins(200×). The expression levels of IRS2, PI3K, AKT, P-AKT, and GLUT4 protein in the NCG were significantly higher than those measured in the MCG (all P < 0.01). In the groups treated with DCI, these levels were significantly higher than those observed in the MCG (all P < 0.01). The expression level of GSK3β protein in the NCG was significantly lower than that reported in the MCG (P < 0.01). Moreover, this level was significantly lower in the DCI-treated groups versus the MCG (P < 0.01).

#### Determination of the Effects of DCI on the Expression Levels of Insulin PI3K/AKT/GSK3β/GLUT4 Signaling Pathway-Related Proteins in Liver Tissues Through Western Blotting

**Figure 6:**
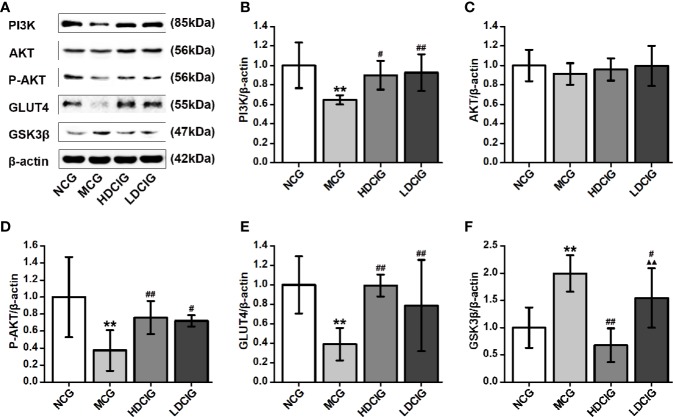
Effects of D-chiro-inositol (DCI) on the expression level of PI3K/AKT/GLUT4/GSK3β signal pathway-related proteins in the liver tissues of db/db mice. **(A)** Representative immunoblots of phosphatidylinositol 3–kinase (P13K), protein kinase B (AKT), phosphor-AKT (S473) (P-AKT), glucose transporters 4 (GLUT4), glycogen synthase kinase 3β (GSK3β), and β-actin are listed. Our samples were derived from the same experiment and blots were processed in parallel. **(B)** The result of PI3K protein expression. **(C)** The result of AKT protein expression. **(D)** The result of P-AKT protein expression. **(E)** The result of GLUT4 protein expression. **(F)** The result of GSK3β protein expression. Grayscale value was determined using Image J based on immunoblot bands (n=3). The results obtained for the different groups were normalized to those reported in the normal control group (NCG). All values are expressed as means ± SD. **P < 0.01 vs. (NCG) mice; ^#^P < 0.05, ^##^P < 0.01 vs. model control group (MCG) mice; ^▲▲^P < 0.01 vs. high-dose D-chiro-inositol group (HDCIG) mice.

The results obtained for the different groups were normalized to those reported in the NCG. As shown in **Figure 6**, the expression levels of PI3K, P-AKT, and GLUT4 proteins in the diabetic model mice were lower than those noted in the normal mice. In contrast, the expression level of GSK3β protein was higher in the liver tissue of diabetic model mice (P < 0.01). Following treatment with DCI, the expression levels of PI3K, P-AKT, and GLUT4 proteins in the liver tissue of db/db mice were upregulated, whereas the expression level of GSK3β protein was downregulated to various degrees (P < 0.05). Moreover, there was no statistical difference in the expression level of AKT protein between the groups.

#### Determination of the Effects of DCI on the Expression Levels of Insulin IRS2/PI3K/AKT/GSK3β/GLUT4 Signaling Pathway-Related mRNA in Liver Tissues Through PCR

**Figure 7:**
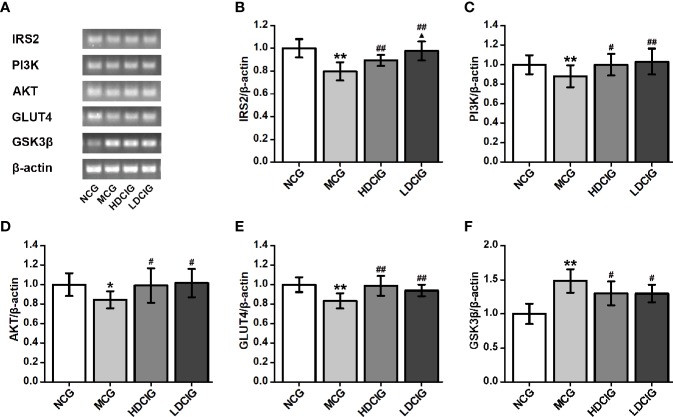
Effects of D-chiro-inositol (DCI) on the expression level of IRS2/PI3K/Akt/GLUT4/GSK3β signal pathway-related mRNA in liver tissues of db/db mice. **(A)** Nucleic acid results of insulin receptor substrate 2 (IRS2), phosphatidylinositol 3–kinase (PI3K), protein kinase B (AKT), glucose transporters 4 (GLUT4), glycogen synthase kinase 3β (GSK3β), and β-actin are listed. Our samples were derived from the same experiment and that gels were processed in parallel. **(B)** The result of IRS2. **(C)** The result of PI3K. **(D)** The result of AKT. **(E)** The result of GLUT4. **(F)** The result of GSK3β. Grayscale value was determined using Image J based on immunoblot bands (n=3). The results obtained for the different groups were normalized to those reported in normal control group (NCG). All values are expressed as means ± SD. *P < 0.05, **P < 0.01 vs. (NCG) mice; ^#^P < 0.05, ^##^P < 0.01 vs. model control group (MCG) mice; ^▲^P < 0.05 vs. high-dose D-chiro-inositol group (HDCIG) mice.

The internal reference was β-actin. The results obtained for the different groups were also normalized to those reported in the NCG. As shown in **Figure 7**, the expression levels of IRS2, PI3K, AKT, and GLUT4 mRNA in liver tissues of NCG mice were higher than those observed in the MCG, which of HDCIG and LDCIG were upregulated (P < 0.05). Moreover, the expression level of GSK3β mRNA in the liver tissue of db/db mice was higher than that reported in the NCG, and this expression level was downregulated after treatment with DCI (P < 0.05).

### *In Vitro* Study

#### Effects of Different Concentrations of DCI on the Viability of HepG2 Cells

**Figure 8:**
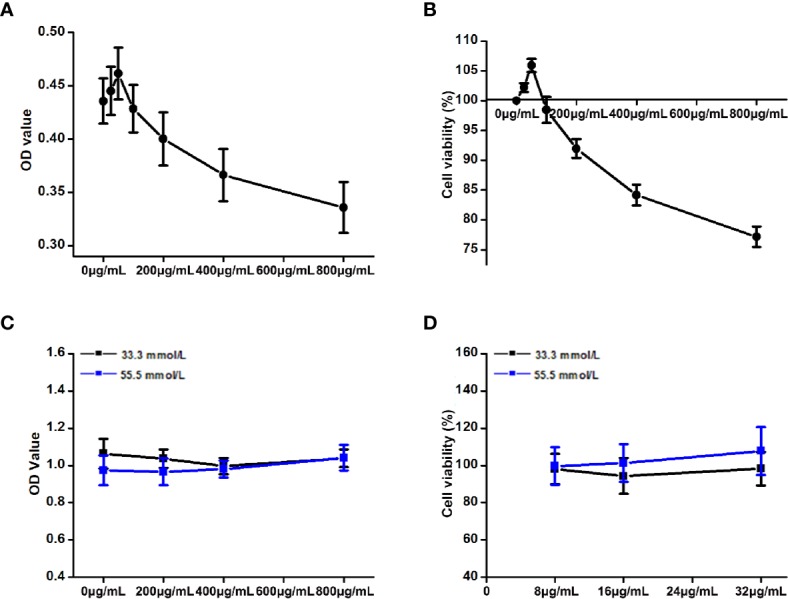
Effects of the different concentrations of D-chiro-inositol (DCI) on the proliferation activity of high glucose-stimulating HepG2 cells. **(A, B)** OD value (the absorbance at 450 nm) of HepG2 cells was detected through the CCK-8 method, and the corresponding cell viability was calculated (%). The effects of the different concentrations of D-chiro-inositol on proliferation activity of Hepg2 cells were calculated (n=6). Cell viability (%) = (OD value of the drug group/OD value of the control group) ×100%. **(C, D)** OD value of high glucose- stimulating and non-high glucose-stimulating HepG2 cells was detected using the CCK-8 method, and the corresponding cell viability was calculated (%) (n=8). All values are expressed as means ± SEM.

The OD values and cell viability results of HepG2 cells treated with different concentrations of DCI are shown in **Figures 8A, B**. When the concentration of DCI ranged from 0 to 50 μg/ml, the cell viability increased to its highest value. Notably, the cell viability decreased as the concentration of DCI increased.

#### Effects of DCI on the Viability of High Glucose-Stimulating HepG2 Cells

The OD values and cell viability results of high glucose-stimulating (55.5 mmol/L) and non–high glucose-stimulating (33.3 mmol/L) HepG2 cells are shown in **Figures 8C, D**. The cell viability of high glucose-stimulating HepG2 cells was higher than that observed in non–high glucose-stimulating cells; however, the difference was not statistically significant.

#### Effects of DCI on Glucose Consumption in High Glucose-Stimulating HepG2 Cells

**Table 3** shows that the glucose consumption in high glucose-stimulated HepG2 cells was significantly higher than that reported in non–high glucose-stimulated cells (normal cells). The glucose consumption of the group of high glucose stimulated by DCI was significantly higher than that observed in the high-glucose control group. In particular, the ratio of glucose consumption to cell proliferation activity (CCK-8) was greater in DCI intervention with a certain concentration effect.

**Table 3:** Effect of different D-chiro-inositol (DCI) concentrations on glucose consumption in HepG2 stimulated by high glucose.

Group	Glucose consumption (mmol/L)	CCK-8 (OD volue)	Glucose consumption/CCK-8
Normal control	7.24 ± 0.649	0.440 ± 0.035	16.596 ± 2.369
High-glucose control	26.139 ± 1.686**	0.518 ± 0.025**	54.482 ± 4.113**
DCI 32 μg/ml	31.843 ± 2.495^##^	0.498 ± 0.011	63.911 ± 5.300^##^
DCI 16 μg/ml	29.714 ± 2.036^##^	0.493 ± 0.034	60.226 ± 5.570^##^
DCI 8 μg/ml	28.842 ± 2.420^##^	0.514 ± 0.028	56.100 ± 5.417^#^

**P < 0.01 vs. Normal control group; ^#^P < 0.05. ^##^P < 0.01 vs. High-glucose control group. (x ± SEM, n=8).

#### Effects of DCI on the Expression Level of IRS2 Protein in HepG2 Cells

**Figure 9:**
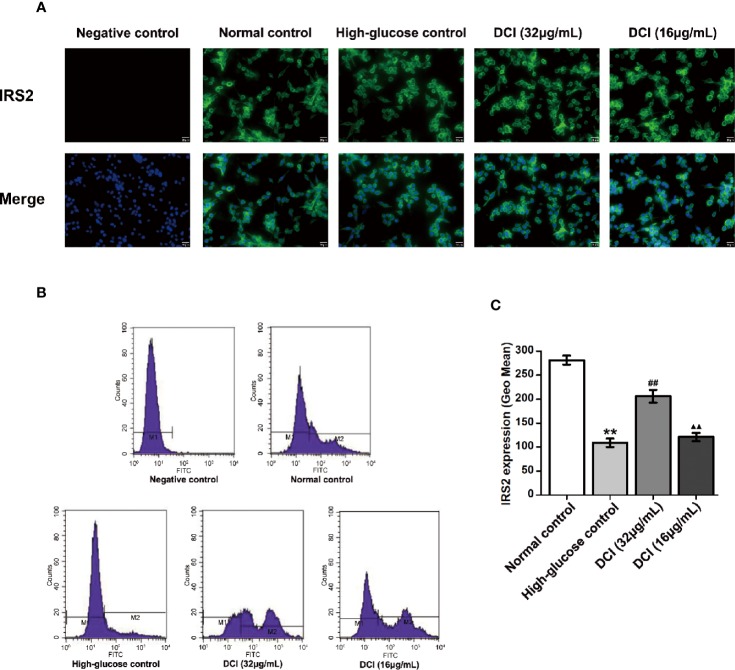
Effects of D-chiro-inositol (DCI) on the expression level of IRS2 protein in high glucose-stimulating HepG2 cells. **(A)** Representative images of fluorescent staining regarding insulin receptor substrate 2 (IRS2) in high glucose- stimulating HepG2 cells in different groups are listed (200×). **(B)** Flow cytometry results regarding IRS2 protein in high glucose-stimulating HepG2 cells in different groups were determined. **(C)** Flow cytometry results regarding IRS2 protein in high glucose-stimulating HepG2 cells in different groups were accounted. All values are expressed as means ± SEM, n=3. **P < 0.01 vs. normal control group cells; ^##^P < 0.01 vs. high-glucose control group cells; ^▲▲^P < 0.01 vs. 32 µg/mL DCI group cells.

The expression level of IRS2 protein in HepG2 cells was detected through staining with green fluorescence (**Figure 9A**). In normal control cells, the expression level of IRS2 protein was higher than that reported in high-glucose control cells. After treatment with DCI, the expression level of IRS2 protein was increased comparing with that observed in high-glucose control cells. The flow cytometry results showed a significant difference between these groups (**Figures 9B, C**).

## Discussion

T2DM accounts for > 90% of diabetes cases, and is characterized by hyperglycemia (Chadha and Morris, 2015; Li et al., 2018). It has been reported that IR is a crucial factor in the development of T2DM, playing an important role in many diseases, such as cardiovascular disease, lipid metabolism, and obesity (Bjornshave and Hermansen, 2014). Furthermore, inhibition or attenuation of the insulin signal is the main cause of IR (Li et al., 2018). The liver is one of the main targets of insulin action, and plays an important role in the development of T2DM (Cai et al., 2018). Insulin signal transduction is associated with IRS2, PI3K, AKT, GSK3β, GLUT4, and other pathway proteins (Schultze et al., 2012). Congenital and acquired factors (e.g., genetic defects, gene mutations, obesity, and the environment) affect the IRS2/PI3K/AKT/GSK3β/GLUT4 pathway, causing IR (Maull et al., 2012). The db/db mice are spontaneous T2DM mice exhibiting very similar symptoms to those observed in clinical T2DM cases (Yin et al., 2018). IR-HepG2 cells are common hepatocyte models for the investigation of the hypoglycemic effects of drugs (Chen et al., 2018). In the present study, we investigated the effect of DCI on glucose metabolism, as well as the mechanism involved in this process.

Previous studies reported that, at a dose range of 35–75 mg/kg, DCI exerts a hypoglycemic effect on diabetic mice (Fan et al., 2018). Therefore, we selected 35 mg/kg and 70 mg/kg as the experimental doses in this study. The duration of the entire intervention was 6 weeks. Our results showed that, in db/db mice, the levels of blood glucose after glucose loading were significantly increased, accompanied by the occurrence of IR. In addition, the level of nonfasting blood glucose in db/db mice fluctuated greatly within 24 h. Notably, the level of blood glucose was higher between 20:00–24:00, whereas it was relatively low during the other time points. However, the overall level of blood glucose was high. Collectively, these results displayed the characteristics of T2DM, which were consistent with those previously reported in the literature (Xie et al., 2002; Michael et al., 2006). The hypoglycemic effect of high- and low-dose DCI on the level of fasting blood glucose was not obvious. However, all concentrations of DCI significantly improved glucose tolerance, stabilized the level of blood glucose within 24 h, and significantly lowered the level of blood glucose versus that observed in the MCG. These observations indicated that DCI exerted a stable hypoglycemic effect. In addition, DCI significantly promoted glucose consumption in HepG2 cells stimulated by high glucose, and increased the ratio of glucose consumption to cell proliferation activity (removing the interference of cell proliferation). The observed increase of glucose consumption was more pronounced in cells treated with DCI, showing a concentration-dependent relationship in the range of 8–32 μg/ml. This result further proved that DCI can promote glucose metabolism.

INS, GSP, and AGEs in the serum are important indicators for assessing the body's abnormal glucose metabolism. We measured these factors to assess the prevalence of diabetes in db/db mice, and then to evaluate the hypoglycemic effect of DCI. The results showed that there was no significant difference in the level of INS between the groups, suggesting that DCI had no effect on the release of INS. However, DCI decreased the levels of GSP, AGEs in the serum of db/db mice, and the effect of high-dose DCI was more pronounced. Furthermore, the level of GSP can reflect the level of blood glucose within 1–3 weeks, which is an important indicator for monitoring the blood glucose (Morris et al., 1986). Sustained high concentrations of blood glucose and various proteins in the body produce nonenzymatic glycation reactions to form AGEs (Gawandi et al., 2018). This process plays an important role in the pathogenesis of chronic complications of diabetes. This study revealed that DCI can reduce the levels of GSP, AGEs in the serum of db/db mice, which may be related to its hypoglycemic effect. Further research is warranted to identify other factors involved in the process.

Hepatic glycogen is the stored form of sugar; its synthesis increases when the blood glucose level is high (Zahra et al., 2013). On the contrary, when the level of blood glucose is low, hepatic glycogen is converted into glucose to supplement blood glucose. Therefore, the synthesis and decomposition of hepatic glycogen is very important in maintaining the relative stability of blood glucose levels (Martin et al., 2004). **Figure 4** showed that the content of glycogen in diabetic model mice was lower than that observed in normal mice. Moreover, the level of hepatic glycogen in mice treated with high- and low-dose DCI was increased compared with that measured in the model group mice. These findings revealed that DCI promotes the synthesis of hepatic glycogen, which may also be one of the mechanisms related to its hypoglycemic effect. In previous studies, obvious liver damage (i.e., steatosis and fibrosis) was found in db/db mice, which may explain the reduced glycogen synthesis.

The role of DCI in the body may be multifaceted, multichanneled, and multitargeted. According to the available evidence, the hypoglycemic mechanism of DCI had been preliminarily explored from the perspective of the insulin signaling pathway (Gao et al., 2016b), suggesting that DCI may upregulate the expression levels of PI3K, P-AKT, and GLUT4, and downregulate the expression of GSK3β through the PI3K/AKT signaling pathway. Our findings are basically consistent with the current findings. According to the classic insulin signal transduction pathway, the mRNA and protein expression levels of several relevant signal factors were detected using PCR and western blotting. The results showed that DCI increased the mRNA and protein expression of IRS2, PI3K, AKT, and GLUT4, upregulated the level of P-AKT protein, and downregulated the level of GSK3β protein in the liver tissues of db/db mice. Furthermore, the expression of fluorescently labeled IRS2 protein in HepG2 cells was detected through flow cytometry. The results showed that DCI upregulated the expression level of IRS2 protein. It is suggested that DCI may increase the expression level of relevant proteins of the insulin PI3K/AKT/GSK3β/GLUT4 signaling pathway to enhance its signal transduction, and improve the functional level of GLUT4 protein. Consequently, this enhances the ability to transport glucose in cells and lower the level of blood glucose.

As an important signaling factor in the insulin signaling pathway, IRS2 is involved in liver metabolism, as well as the development and survival of pancreatic parenchyma cells for hepatic metabolism and pancreatic beta-cells (Joana Moitinho et al., 2014; Saltiel, 2016). Studies have shown that IRS2 may inhibit hyperglycemia and hyperinsulinemia, while promoting endogenous glucose production and glycogen synthesis (Previs et al., 2000; Bertinat et al., 2018).

Recent studies have shown that IR is a key factor for the occurrence and development of T2DM (Nuthalapati and Indukuri, 2016), and a major feature of T2DM (Zahra et al., 2013). As the target organ of insulin action, IR in the liver is a main component of the mechanism of T2DM (Polyzos et al., 2012). The mechanism of IR mainly involves IRS2, PI3K, GLUT4, and other pathway proteins. Therefore, in this study, the effect of DCI on the IR in the liver of db/db diabetic mice and HepG2 cells was investigated through the insulin IRS2/PI3K/GLUT4 signaling pathway. Furthermore, the mechanism of IR reduction by DCI was investigated at the cellular and molecular levels, providing the basis for the development into hypoglycemic drugs and clinical application of DCI.

## Conclusions

Overall, DCI may significantly decrease and stabilize the level of blood glucose, improve glucose tolerance, and reduce IR in spontaneous type 2 diabetic db/db mice. The hypoglycemic mechanism of DCI involves the following: increase in the mRNA and protein expression levels of IRS2, PI3K, AKT, and GLUT4 signal factors; promotion of phosphorylation of AKT proteins, downregulation of the expression level of GSK3β, increase of glycogen synthesis, and promotion of the signal transduction of insulin PI3K/AKT/GSK3β/GLUT4 pathway. However, the action mechanism of DCI may involve multiple targets and pathways. Therefore, further research is warranted to elucidate this mechanism.

## Data Availability Statement

The raw data supporting the conclusions of this article will be made available by the authors, without undue reservations, to any qualified researcher.

## Ethics Statement

All procedures of animal experiments were approved by the Animal Ethics Committee of North China University of Science and Technology, according to the guidelines established by the European Union (Directive 2010/63/EU for animal experiments) and the National Institute of Health of the USA (NIH Publications No. 8023, revised 1978).

## Author Contributions

SH and JB designed the study. CF, WL, and MW performed the experiments. CF, WL, and SH made statistical analysis. CF, WL, and XG wrote the manuscript. CF, SH, and JB revised the final draft of the manuscript.

## Funding

This work was supported by the Natural Science Foundation of Hebei Province (grant number: H2014209194).

## Conflict of Interest

The authors declare that the research was conducted in the absence of any commercial or financial relationships that could be construed as a potential conflict of interest.

## Supplementary Material

Data Sheet 1 The figures of original WB results, including β-actin, PI3K, AKT, P-AKT, GLUT4, and GSK3β.

Data Sheet 2 The figures of original PCR results, including β-actin, IRS2, PI3K, AKT, GLUT4, and GSK3β.

## References

[B1] Bao S. Y. Han S. Y. Cheli-geer Chao-riya Ao W.-L. (2016). Effects of agiophyllum oligo saccharides on insulin resistance of Goto-Kakizaki rats. Chin. Pharmacol. Bull. v.32 (03), 403–409. 10.3969/j.issn.1001-1978.2016.03.021

[B2] Bertinat R. Westermeier F. Silva P. Gatica R. Oliveira J. M. Nualart F. (2018). The Antidiabetic Agent Sodium Tungstate Induces Abnormal Glycogen Accumulation in Renal Proximal Tubules from Diabetic IRS2-Knockout Mice. J. Diabetes Res. 2018, 1–10. 10.1155/2018/5697970 PMC5996472 30003110

[B3] Bjornshave A. Hermansen K. (2014). Effects of dairy protein and fat on the metabolic syndrome and type 2 diabetes. Rev. Diabetes Stud. 11 (2), 153–166. 10.1900/RDS.2014.11.153 PMC4310065 25396403

[B4] Cai F. Liu J. R. Fan D. M. Shen J. X. (2018). Roles of TNFa, interleukin and other cytokines in the type 2 diabetes mellitus and nonalcoholic fatty liver disease. J. Shenyang Med. College. 020 (002), 162–165. 10.16753/j.cnki.1008-2344.2018.02.019

[B5] Chadha G. S. Morris M. E. (2015). Effect of Type 2 Diabetes Mellitus and Diabetic Nephropathy on IgG Pharmacokinetics and Subcutaneous Bioavailability in the Rat. AAPS J. 17 (4), 1–11. 10.1208/s12248-015-9771-3 25924888 PMC4477001

[B6] Chen Y. Xu G. L. Li B. T. L. I. Jiang L. Sheng Y. X. (2018). Effect of Gegen Qinlian Tang Serum in Regulating Glucose Metabolism of IR-HepG2 Cells. Chin. J. Exp. Tradit. Med. Formulae. 024 (010), 156–160. 10.13422/j.cnki.syfjx.20180836

[B7] Cheung L. T. F. Chan R. S. M. Ko G. T. C. Lau E. S. H. Chow F. C. C. Kong A. P. S. (2018). Diet quality is inversely associated with obesity in Chinese adults with type 2 diabetes. Nutr. J. 17 (1), 63. 10.1186/s12937-018-0374-6 29970112 PMC6031190

[B8] Cordain L. Eades M. R. Eades M. D. (2003). Hyperinsulinemic diseases of civilization: more than just Syndrome X. Comp. Biochem. Physiol. Part A Mol. Integr. Physiol. 136 (1), 95–112. 10.1016/S1095-6433(03)00011-4 14527633

[B9] Fan C. X. Wei M. Zhang D. D. Gao Q. Y. Huang H. X. Wang J. X. (2018) Effect of D-chiro-inositol on hypoglycemic and liver protection in type 2 diabetic db/db mice and its mechanism. Chin. Pharmacol. Bull. 34 (12), 90–95. 10.3969/j.issn.1001-1978.2018.12.018

[B10] Gao H. L. Song Y. Wang P. Fu M. (2016a). Effects of D-chiro-inositol on glucose consumption in IR-HepG2 cells. Acta Academiae Med. Qingdao Universitatis. (06), 5–7. 10.13361/j.qdyxy.201606001

[B11] Gao Y. F. Zhang M. N. Wang T. X. Wu T. C. Ai R. D. Zhang Z. S. (2016b). Hypoglycemic effect of D-chiro-inositol in type 2 diabetes mellitus rats through the PI3K/Akt signaling pathway. Mol. Cell. Endocrinol. 433 (C), 26–34. 10.1016/j.mce.2016.05.013 27212205

[B12] Gartmann L. Wex T. Grüngreiff K. Reinhold D. Kalinski T. Malfertheiner P. (2018). Expression of Zinc transporters ZIP4, ZIP14 and ZnT9 in hepatic carcinogenesis - an immunohistochemical study. J. Trace Elements Med. Biol. 49, 35–42. 10.1016/j.jtemb.2018.04.034 29895370

[B13] Gawandi S. Gangawane S. Chakrabarti A. Kedare S. Bantwal K. Wadhe V. (2018). A Study of Microalbuminuria (MAU) and Advanced Glycation End Products (AGEs) Levels in Diabetic and Hypertensive Subjects. Indian J. Clin. Biochem. 33 (Suppl 1), 1–5. 10.1007/s12291-017-0638-5 29371774 PMC5766458

[B14] Gou X. Wang W. Zou S. Qi Y. Xu Y. (2018). Protein kinase C epsilon mediates the inhibition of angiotensin II on the slowly activating delayed-rectifier potassium current through channel phosphorylation. J. Mol. Cell. Cardiol. 116, 165–174. 10.1016/j.yjmcc.2018.02.010 29452158

[B15] Hummel K. P. Dickie M. M. Coleman D. L. (1966). Diabetes, a new mutation in the mouse. Science 153 (3740), 1127–1128. 10.1126/science.153.3740.1127 5918576

[B16] Ji Y. K. Lee S. H. Song E. H. Park Y. M. Lim J. Y. Kim D. J. (2009). A critical role of STAT1 in streptozotocin-induced diabetic liver injury in mice: Controlled by ATF3. Cell. Signal. 21 (12), 1758–1767. 10.1016/j.cellsig.2009.07.011 19647793 PMC4779502

[B17] Joana Moitinho O. Rebuffat S. A. Rosa G. Burks D. J. Ainhoa G. Kalko S. G. (2014). Tungstate promotes β-cell survival in Irs2-/- mice. Am. J. Physiol. Endocrinol. Metab. 306 (1), E36. 10.1152/ajpendo.00409.2013 24253047

[B18] Ken-Ichi Y. Masanori Y. Tetsuro M. Maya I. Masaki K. Hitoshi A. (2006). Genetic modification of Bacillus subtilis for production of D-chiro-inositol, an investigational drug candidate for treatment of type 2 diabetes and polycystic ovary syndrome. Appl. Environ. Microbiol. 72 (2), 1310–1315. 10.1128/AEM.72.2.1310-1315.2006 16461681 PMC1392952

[B19] Larner J. Brautigan D. L. Thorner M. O. (2010). D-chiro-inositol glycans in insulin signaling and insulin resistance. Mol. Med. 16 (11–12), 543–552. 10.2119/molmed.2010.00107 20811656 PMC2972396

[B20] Li J. Feng J. Wei H. Liu Q. Yang T. Hou S. (2018). The Aqueous Extract of Gynura divaricata (L.) DC. Improves Glucose and Lipid Metabolism and Ameliorates Type 2 Diabetes Mellitus. Evidence-Based Complement. Altern. Med. 2018 (5), 1–11. 10.1155/2018/8686297 PMC5828177 29599810

[B21] Liang W. S. Zhang D. D. Kang J. L. Meng X. B. Yang J. B. Yang L. (2018). Protective effects of rutin on liver injury in type 2 diabetic db/db mice. Biomed. Pharmacother. 107, 721–728. 10.1016/j.biopha.2018.08.046 30138894

[B22] Martin K. Attila B. Elisabeth B. Christian A. Peter N. Chiara D. M. (2004). Alterations in postprandial hepatic glycogen metabolism in type 2 diabetes. Diabetes 53 (12), 3048–3056. 10.2337/diabetes.53.12.3048 15561933

[B23] Maull E. A. Habibul A. Joshua E. Longnecker M. P. Ana N. A. Jingbo P. (2012). Evaluation of the association between arsenic and diabetes: a National Toxicology Program workshop review. Environ. Health Perspect. 120 (12), 1658–1670. 10.1289/ehp.1104579 22889723 PMC3548281

[B24] Mehanna E. T. Barakat B. M. Elsayed M. H. Tawfik M. K. (2018). An optimized dose of raspberry ketones controls hyperlipidemia and insulin resistance in male obese rats: effect on adipose tissue expression of adipocytokines and Aquaporin 7. Eur. J. Pharmacol. 832, 81–89. 10.1016/j.ejphar.2018.05.028 29787773

[B25] Michael L. Martin B. Mark R. Werner W. U. Stulnig T. M. (2006). Blood glucose-lowering nuclear receptor agonists only partially normalize hepatic gene expression in db/db mice. J. Pharmacol. Exp. Ther. 316 (2), 797–804. 10.1124/jpet.105.093831 16260581

[B26] Morris M. A. Grandis A. S. Litton J. C. (1986). Longitudinal assessment of glycosylated blood protein concentrations in normal pregnancy and gestational diabetes. Diabetes Care 9 (2), 107–110. 10.2337/diacare.9.2.107 3698777

[B27] Nuthalapati R. K. Indukuri B. R. (2016). Association between glycemic control and morning blood surge with vascular endothelial dysfunction in type 2 diabetes mellitus patients. Indian J. Endocrinol. Metab. 20 (2), 182–188. 10.4103/2230-8210.176349 27042413 PMC4792018

[B28] Polyzos S. A. Kountouras J. Deretzi G. Zavos C. Mantzoros C. S. (2012). The emerging role of endocrine disruptors in pathogenesis of insulin resistance: a concept implicating nonalcoholic fatty liver disease. Curr. Mol. Med. 12 (1), 68–82. 10.2174/156652412798376161 22082482

[B29] Previs S. F. Withers D. J. Ren J. M. White M. F. Shulman G. I. (2000). Contrasting effects of IRS-1 versus IRS-2 gene disruption on carbohydrate and lipid metabolism in vivo. J. Biol. Chem. 275 (50), 38990–38994. 10.1074/jbc.M006490200 10995761

[B30] Rutter M. K. Mccomb J. M. Brady S. Marshall S. M. (1999). Silent myocardial ischemia and microalbuminuria in asymptomatic subjects with non-insulin-dependent diabetes mellitus. Am. J. Cardiol. 83 (1), 27–31. 10.1016/S0002-9149(98)00777-2 10073780

[B31] Saltiel A. R. (2016). Insulin Signaling in the Control of Glucose and Lipid Homeostasis. Handb. Exp. Pharmacol. 233, 51–71. 10.1007/164_2015_14 26721672

[B32] Schultze S. M. Hemmings B. A. Niessen M. Tschopp O. (2012). PI3K/AKT, MAPK and AMPK signalling: protein kinases in glucose homeostasis. Expert Rev. Mol. Med. 14, e1. 10.1017/S1462399411002109 22233681

[B33] Shridas P. De Beer M. C. Webb N. R. (2018). High-density lipoprotein inhibits serum amyloid A-mediated reactive oxygen species generation and NLRP3 inflammasome activation. J. Biol. Chem. 293 (34), 13257–13269. 10.1074/jbc.RA118.002428 29976759 PMC6109913

[B34] Soll J. M. Brickner J. R. Mudge M. C. Mosammaparast N. (2018). RNA ligase-like domain in activating signal cointegrator 1 complex subunit 1 (ASCC1) regulates ASCC complex function during alkylation damage. J. Biol. Chem. 293 (35), 13524–13533. 10.1074/jbc.RA117.000114 29997253 PMC6120213

[B35] Song Y. Zou L. Zhao J. L. Peng L. X. Wei L. I. Wang X. P. (2016). The change of D-chiro inositol content in tartary buckwheat during germination process. Food Sci. Technol. (2), 80–83. 10.13684/j.cnki.spkj.2016.02.015

[B36] Steadman K. J. Burgoon M. S. Schuster R. L. Lewis B. A. Edwardson S. E. Obendorf R. L. (2000). Fagopyritols, D-chiro-inositol, and other soluble carbohydrates in buckwheat seed milling fractions. J. Agric. Food Chem. 48 (7), 2843–2847. 10.1021/jf990709t 10898633

[B37] Tian W. Chen L. Zhang L. Wang B. Li X. B. Fan K. R. (2017). Effects of ginsenoside Rg1 on glucose metabolism and liver injury in streptozotocin-induced type 2 diabetic rats. Genet. Mol. Res. 16 (1), gmr16019463. 10.4238/gmr16019463 28362999

[B38] Whiting L. Danaher R. N. Ruggiero K. Lee C.-C. Chaussade C. Mulvey T. (2013). D-chiro-inositol attenuates epinephrine-stimulated hepatic glucose output in the isolated perfused liver independently of insulin. Hormone Metab. Res. 45 (05), 394–397. 10.1055/s-0032-1330016 23225249

[B39] Xie J. T. Zhou Y. P. Dey L. Attele A. S. Wu J. A. Gu M. (2002). Ginseng berry reduces blood glucose and body weight in. Phytomedicine 9 (3), 254–258. 10.1078/0944-7113-00106 12046868

[B40] Yin J. Wang H. Lu G. (2018). Umbelliferone alleviates hepatic injury in diabetic db/db mice via inhibiting inflammatory response and activating Nrf2-mediated antioxidant. Biosci. Rep. 38 (4), BSR20180444. 10.1042/BSR20180444 29967293 PMC6131207

[B41] Zahra B. Parvin M. Fereidoun A. (2013). Potential efficacy of broccoli sprouts as a unique supplement for management of type 2 diabetes and its complications. J. Med. Food 16 (5), 375–382. 10.1089/jmf.2012.2559 23631497

