# Supplementary material for: Effects of D-Chiro-Inositol on Glucose Metabolism in db/db Mice and the Associated Underlying Mechanisms
Source: Front Pharmacol. 2020 Mar 26;11:354. doi: 10.3389/fphar.2020.00354 (PMC7113635; doi:10.3389/fphar.2020.00354)
Supplement: Data Sheet 1 — The figures of original WB results, including β-actin, PI3K, AKT, P-AKT, GLUT4, and GSK3β. [file DataSheet_1.zip › WB/explanation.docx]

Our application of WB bands:

Bactin: 1-4

PI3K: 1-4

AKT: 1-4

p-AKT: 1-4

GLUT4: 1-4(2)

GSK3β: 1-4
