# Supplementary figures and images for: Effects of D-Chiro-Inositol on Glucose Metabolism in db/db Mice and the Associated Underlying Mechanisms
Source: Front Pharmacol. 2020 Mar 26;11:354. doi: 10.3389/fphar.2020.00354 (PMC7113635; doi:10.3389/fphar.2020.00354)

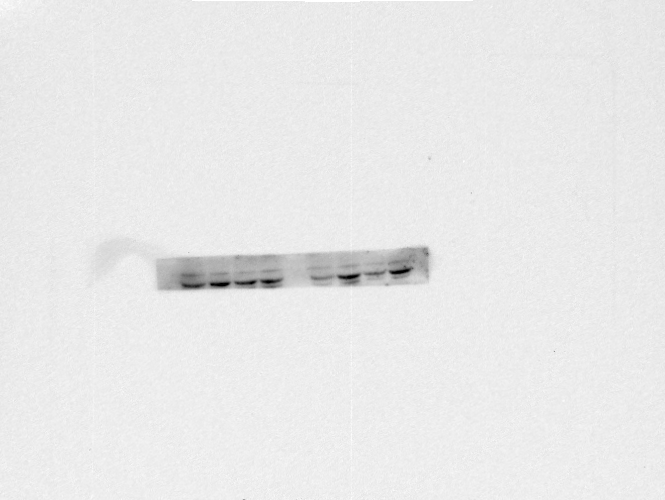

Supplement: Data Sheet 1 — The figures of original WB results, including β-actin, PI3K, AKT, P-AKT, GLUT4, and GSK3β. [file DataSheet_1.zip › WB/2018-0514-L-GSK3a┬.jpg]

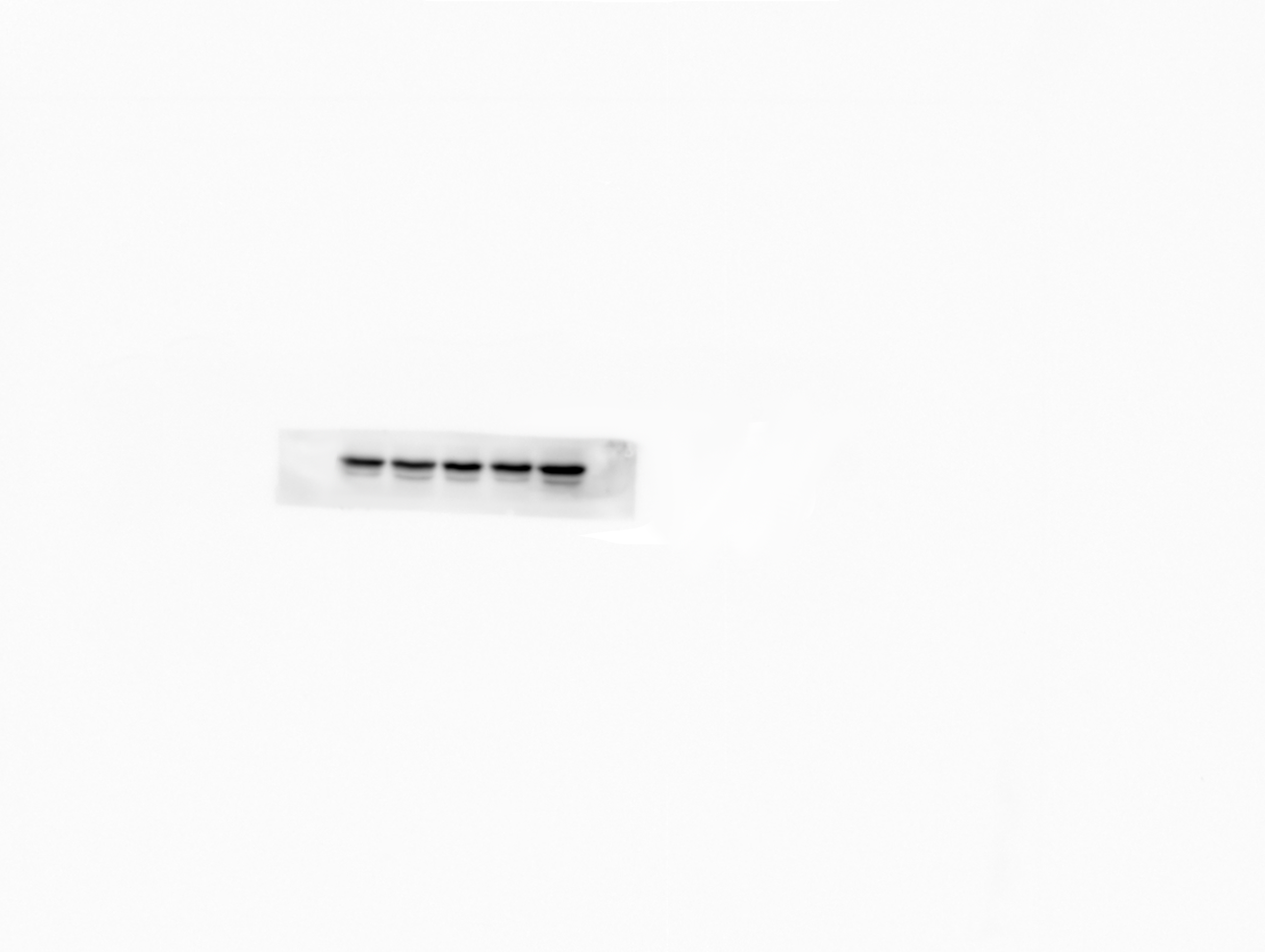

Supplement: Data Sheet 1 — The figures of original WB results, including β-actin, PI3K, AKT, P-AKT, GLUT4, and GSK3β. [file DataSheet_1.zip › WB/2018-0609-L-AKT.tif]

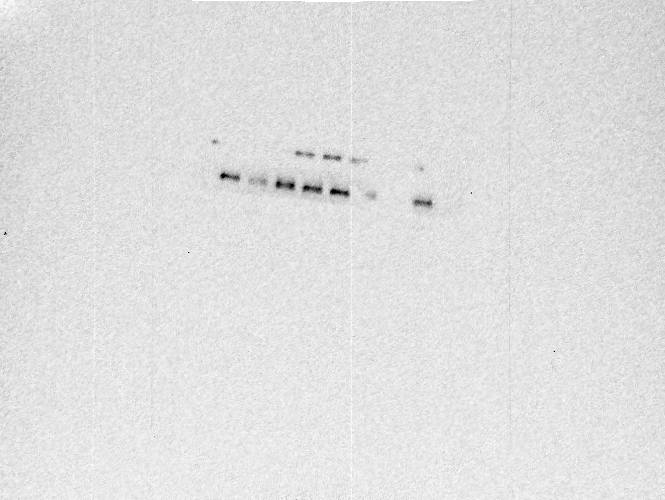

Supplement: Data Sheet 1 — The figures of original WB results, including β-actin, PI3K, AKT, P-AKT, GLUT4, and GSK3β. [file DataSheet_1.zip › WB/2018-0712-L-GLUT4.bmp]

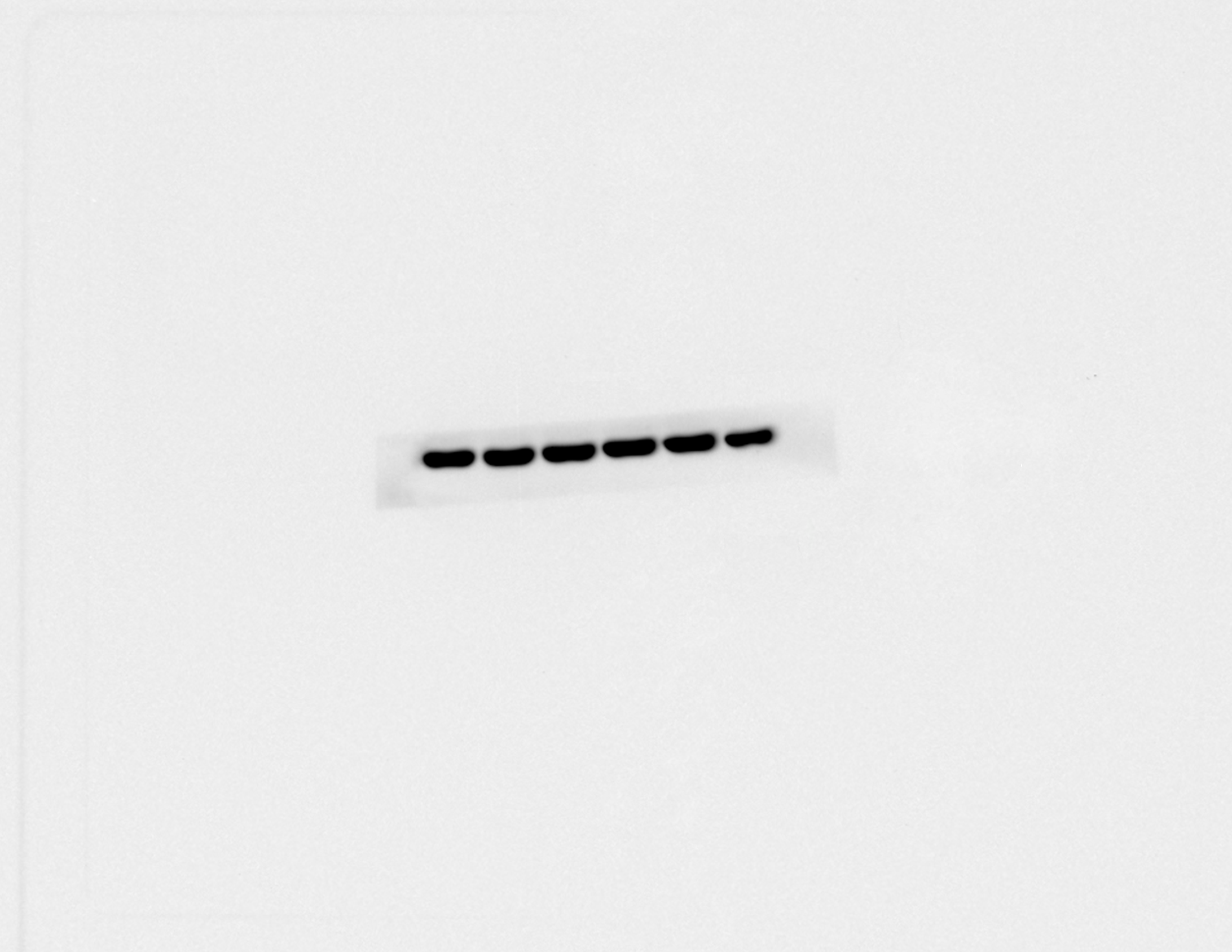

Supplement: Data Sheet 1 — The figures of original WB results, including β-actin, PI3K, AKT, P-AKT, GLUT4, and GSK3β. [file DataSheet_1.zip › WB/2018-0716-L-a┬-actin.tif]

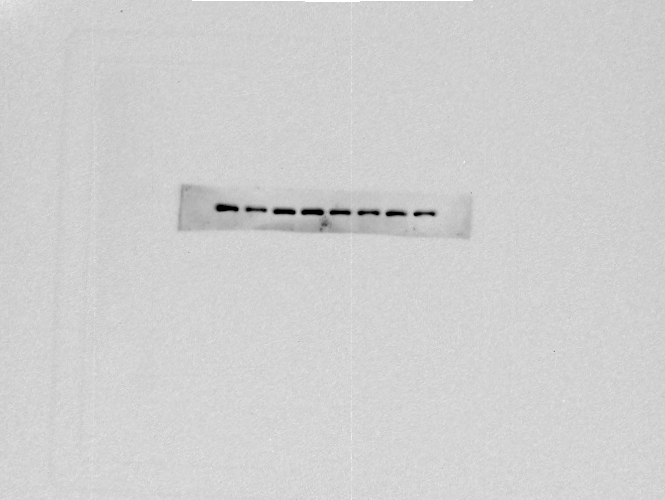

Supplement: Data Sheet 1 — The figures of original WB results, including β-actin, PI3K, AKT, P-AKT, GLUT4, and GSK3β. [file DataSheet_1.zip › WB/2018-0718-L-PI3K.bmp]

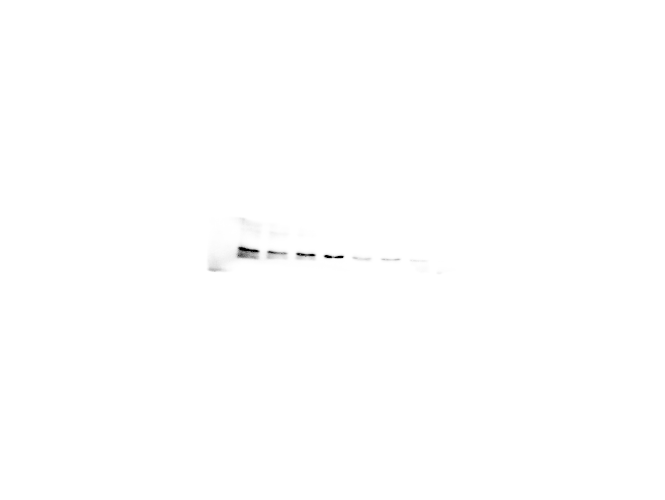

Supplement: Data Sheet 1 — The figures of original WB results, including β-actin, PI3K, AKT, P-AKT, GLUT4, and GSK3β. [file DataSheet_1.zip › WB/2018-0723-L-p-akt.bmp]

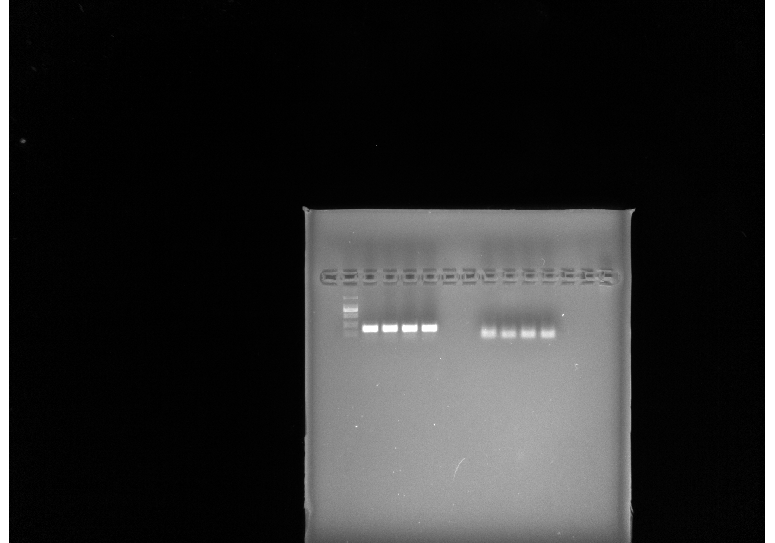

Supplement: Data Sheet 2 — The figures of original PCR results, including β-actin, IRS2, PI3K, AKT, GLUT4, and GSK3β. [file DataSheet_2.zip › pcr/AKT.tif]

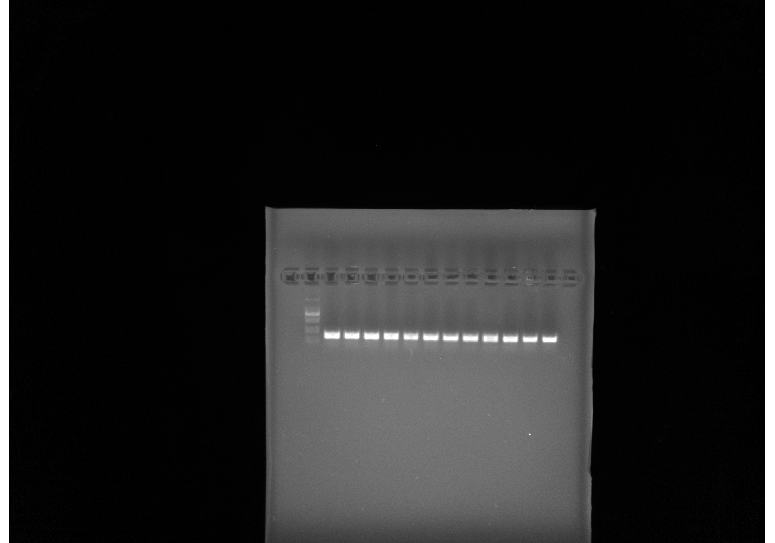

Supplement: Data Sheet 2 — The figures of original PCR results, including β-actin, IRS2, PI3K, AKT, GLUT4, and GSK3β. [file DataSheet_2.zip › pcr/B-actin.tif]

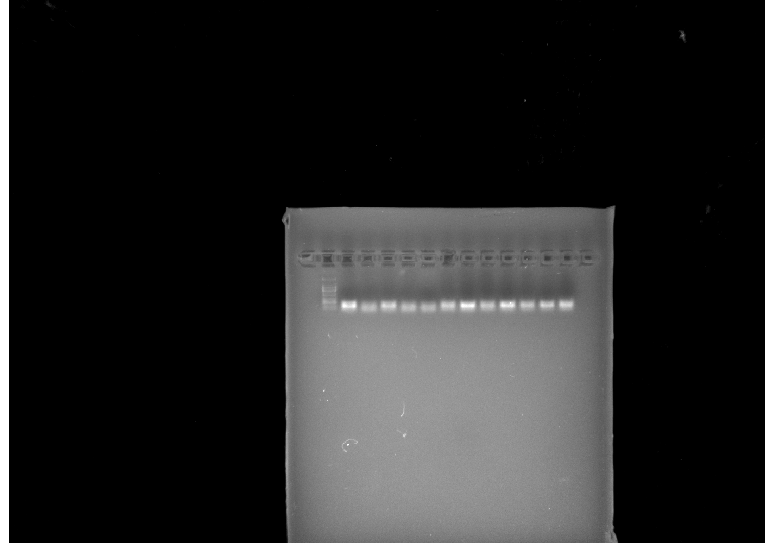

Supplement: Data Sheet 2 — The figures of original PCR results, including β-actin, IRS2, PI3K, AKT, GLUT4, and GSK3β. [file DataSheet_2.zip › pcr/GLUT4.tif]

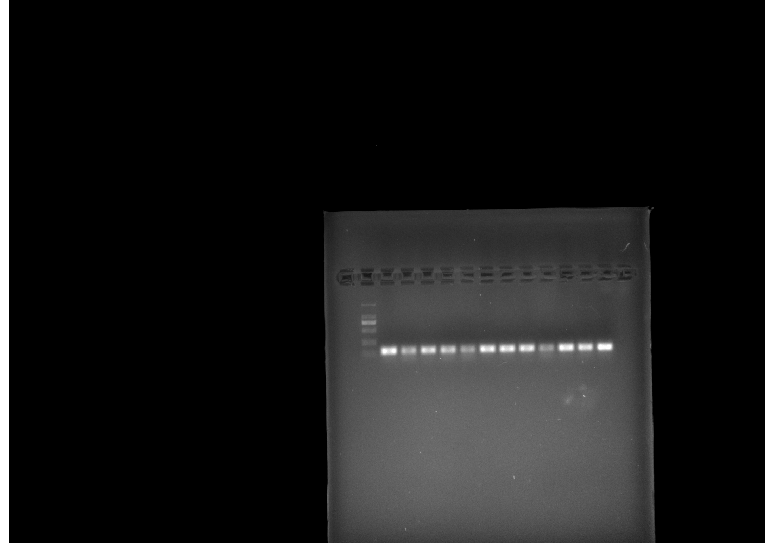

Supplement: Data Sheet 2 — The figures of original PCR results, including β-actin, IRS2, PI3K, AKT, GLUT4, and GSK3β. [file DataSheet_2.zip › pcr/GSK3.tif]

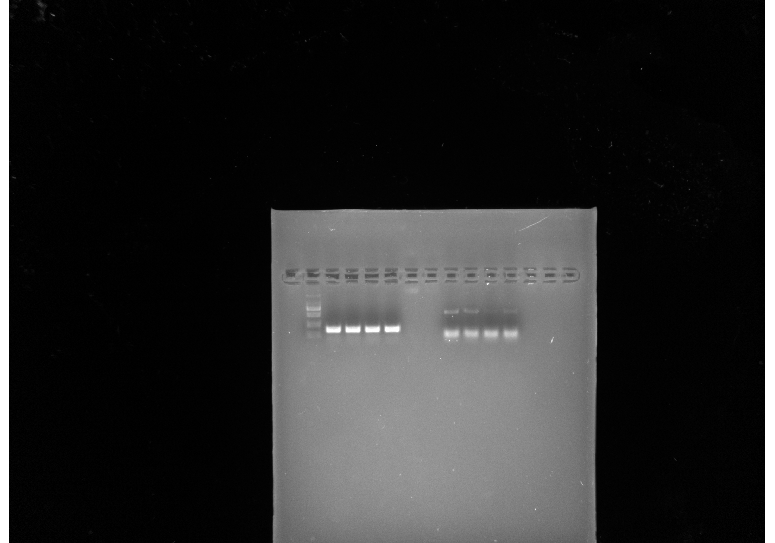

Supplement: Data Sheet 2 — The figures of original PCR results, including β-actin, IRS2, PI3K, AKT, GLUT4, and GSK3β. [file DataSheet_2.zip › pcr/IRS2.tif]

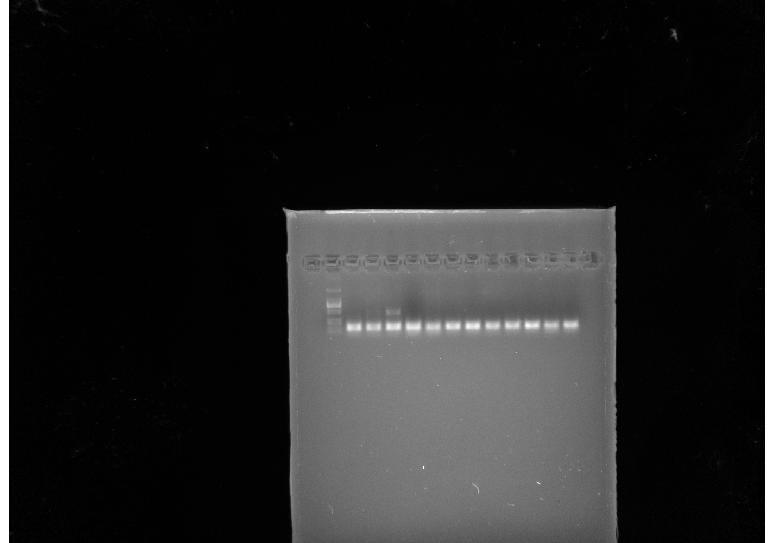

Supplement: Data Sheet 2 — The figures of original PCR results, including β-actin, IRS2, PI3K, AKT, GLUT4, and GSK3β. [file DataSheet_2.zip › pcr/PI3K.tif]
