# Supplementary material for: Effects of D-Chiro-Inositol on Glucose Metabolism in db/db Mice and the Associated Underlying Mechanisms
Source: Front Pharmacol. 2020 Mar 26;11:354. doi: 10.3389/fphar.2020.00354 (PMC7113635; doi:10.3389/fphar.2020.00354)
Supplement: Data Sheet 2 — The figures of original PCR results, including β-actin, IRS2, PI3K, AKT, GLUT4, and GSK3β. [file DataSheet_2.zip › pcr/explanation.docx]

Our application of PCR bands:

Bactin: 1-4

IRS2: 5-8

PI3K: 1-4

AKT: 5-8

GLUT4: 9-12

GSK3β: 5-8
